# Treating PTSD with Imagery Rescripting in underweight eating disorder patients: a multiple baseline case series study

**DOI:** 10.1186/s40337-022-00558-1

**Published:** 2022-03-09

**Authors:** Marieke C. ten Napel-Schutz, Maartje Vroling, Suzanne H. W. Mares, Arnoud Arntz

**Affiliations:** 1 Department of Eating Disorders (Amarum), GGNet Mental Health, Warnsveld, The Netherlands; 2 Radboud Centre Social Science, Radboud University, Nijmegen, The Netherlands; 3 Department of Clinical Psychology, University of Amsterdam, Amsterdam, The Netherlands

**Keywords:** Anorexia nervosa, Underweight, Post traumatic stress disorder, IMagery ReScripting

## Abstract

**Background:**

Eating disorder patients with posttraumatic stress disorder have worse treatment results regarding their eating disorder than patients without posttraumatic stress disorder. Many eating disorder patients with co-morbid posttraumatic stress disorder symptoms are not treated for posttraumatic stress disorder symptoms during an underweight state. We propose that treatment of posttraumatic stress disorder is possible for underweight patients and that their trauma symptoms decrease with the use of Imagery Rescripting. We also investigated whether treatment of trauma influences eating disorder pathology in general and the process of weight gain specifically.

**Method:**

Ten patients in clinical treatment (BMI 14–16.5) participated. A multiple baseline design was used, with baseline varying from 6 to 10 weeks, a 6-week treatment phase, a 3-week follow-up period and a 3-month follow-up measurement. Data were analysed with mixed regression.

**Results:**

Evidence was found that Imagery Rescripting had strong positive effects on posttraumatic stress disorder symptoms without interfering with eating disorder treatment. Positive effects were also found on a range of secondary emotional and cognitive measures.

**Conclusion:**

Imagery Rescripting of traumatic memories is a possible and safe intervention for underweight eating disorder patients. It also had positive clinical effects.

*Trial registration* Netherlands trial register (NTR) Trial NL5906 (NTR6094). Date of registration 09/23/2016. https://www.trialregister.nl/trial/5906.

## Plain English summary

The present study was the first to investigate treatment of posttraumatic stress symptoms, such as re-experiences and flash backs, in underweight eating disorder patients.

This research is important because these patients:

- Often do not receive treatment for trauma symptoms while they are underweight.
- Have worse eating disorder treatment results than patients without posttraumatic stress disorder.

Patients and patient organisations have for years expressed the wish that posttraumatic stress disorder be treated during the weight gaining phase of eating disorder treatment. The results of this study are important because they show that treatment for trauma is possible for patients that are underweight.

Ten patients received trauma treatment. Trauma and eating disorder symptoms were measured before, during and after treatment. We tested whether trauma symptoms were reduced during and after treatment.

This research was done because patients requested posttraumatic stress disorder treatment while they were underweight. Previous patients were involved in the development of the interview and all participants were interviewed about their experiences. The findings will be published and presented at eating disorder conferences attended by patients.

## Background

The rate of Posttraumatic Stress Disorder (PTSD) in clinically admitted patients with Anorexia Nervosa (AN) is estimated to be between 10% [28] and 47% [54]. Generally, AN patients with comorbid PTSD have more severe obsessive–compulsive symptoms, depressed and anxious mood, lower self-esteem, and more interpersonal problems [11, 15, 37]. From clinical practice we learned that AN in patients with PTSD is often more difficult to treat than AN in patients without PTSD. Several studies have found higher relapse rates, poorer treatment response, or more frequent premature termination of treatment in patients with AN and PTSD [15, 48, 49]. Given the complexity PTSD adds to the treatment of AN, it is important to investigate whether the standard treatment of AN patients with co-morbid PTSD symptoms can be expanded on with trauma-treatment.

Despite the difficulty PTSD causes in the treatment of AN, treating trauma in underweight Eating Disorder (uED) patients is a controversial issue for several reasons. First, the assumption exists that being in an underweight state and/or being malnourished suppresses cognitive functioning [1, 17, 35], while this is needed for effective trauma-focused treatment. Because of this the clinical tradition is to not focus on trauma processing during uED treatment. However, Rylander et al. [50] found no significant impairments in cognition in uED patients.

Secondly treating trauma in underweight patients is controversial because trauma-treatment requires sufficient experience of emotions. There are studies that show that people with AN attenuate emotional expression and avoid negative affect [18] leading to the assumption that being underweight results in reduced emotional experience and thus ineffective trauma focused treatment. More so clinicians report reduced emotional experiences because of starvation [16]. However, other studies found that underweight eating disorder patients report the same or elevated levels of negative affect or emotions in comparison to healthy controls, and that negative affect is not related to Body Mass Index (BMI) [18, 56, 59].

The third reason treating trauma during an underweight state is controversial is because trauma-treatment requires the ability to regulate emotions, and research results on emotion regulation skills in underweight patients are inconsistent. Some studies reveal that people with AN have more difficulty regulating emotions than healthy controls [12, 31]. Qualitative research described that AN patients experience their eating disorder as helping them to manage difficult experiences [30, 36]. Also, patients report that not-eating and being underweight helps them to handle negative emotions [33, 41]. Other studies found inconsistent associations or no associations between BMI and the degree of emotion regulation problems [13, 32, 47]. If the emotion regulation problems persist, even with healthy weight, this is not a valid argument for postponing trauma processing (until the patient has a healthy BMI).

With lack of evidence for deteriorated cognitive functioning and inconsistent research results for insufficient experience of emotions and emotion regulation skills, reconsidering the possibility of focusing on trauma processing for uED patients is important. Otherwise, the risk is that trauma remains untreated in this population and that PTSD symptoms will continue to interfere with eating disorder treatment. Also, the assumption that trauma processing is not possible in this population then remains untested.

As The NICE guideline (The NICE guideline, 1.8.12, 2017) indicates, there is little evidence for treatments for patients with an eating disorder and comorbidity, and randomized clinical trials are necessary. Since 2017, the Dutch treatment guidelines for eating disorder treatment recommends starting trauma processing if the symptoms interfere with eating disorder treatment too much (despite patients being underweight). They also recognize that there is not enough evidence for this recommendation (GGZ Zorgstandaard eetstoornissen, 2017) [2]. American guidelines, however, advise against offering psychotherapy to underweight patients [62].

With a lack of evidence for treatment of PTSD in uED patients, the NICE guideline suggests taking the following into account “the severity and complexity of the eating disorder, the comorbidity, the person’s level of functioning and the preference of the patient and if possible, their family members or carers” (The NICE guideline, 1.8.12, 2017) [39]. In the Netherlands it is common practice to offer psychotherapeutic management and/or cognitive therapy during weight restoration, and to start (insight oriented) psychotherapy only after this phase has (almost) been completed. Hence, trauma-related problems (if addressed at all) are only addressed in the later phase of treatment. Given the lack of scientific evidence, and the incoherence in clinical guidelines, it important to examine the effect of trauma treatment in uED patients.

In line with suggestions by Brewerton [11] Dutch Feeding and Eating Disorder (FED) patient organizations have for years requested) that underlying trauma be treated in an earlier phase of eating disorder treatment (IxtaNoa personal communication, May 2012). We therefore investigated whether traumatized patients can indeed be treated for their trauma during their weight restoration phase.

Given the vulnerable uED population it is important to make an informed choice as to which type of trauma treatment should be added to regular eating disorder treatment. Current treatment guidelines for PTSD describe two treatments which are considered equally effective: individual trauma-focused cognitive behavioural therapy with imaginal exposure (IE) and eye movement desensitization and reprocessing (EMDR) (Multidisciplinaire Richtlijn Angststoornissen, 3^e^ revision, 2013(1.0)) [55]. Recently, Imagery Rescripting (IMRS) has emerged as a promising evidence-based treatment for PTSD. An initial randomized controlled trial comparing the combination of IE and IMRS (IE + IMRS) to IE only to treat PTSD demonstrated that, beside a comparable effect on reducing PTSD-related symptoms, IE + IMRS had a superior effect in diminishing other trauma-related emotions such as anger, shame, and guilt [7]. Moreover, treatment dropout was significantly lower in the IE + IMRS condition. A recent pilot study on IMRS also showed low dropout rates, indicating that IMRS might be less aversive to patients [44]. Similarly, an international Randomized Clinical Trial (RCT) comparing IMRS to EMDR demonstrated large effects for both treatments in patients with PTSD due to childhood trauma, with very low dropout [10]. Another RCT demonstrated that a combination of Skills Training in Affect and Interpersonal Regulation (STAIR) and IMRS did not perform better than IMRS alone. Also, IMRS proved to be very effective in the treatment of PTSD due to childhood abuse, and emotion regulation improved more by IMRS than by STAIR [44, 46*Submitted*]. A meta-analysis and review of IMRS concluded that the method is a promising therapeutic technique, with large effects in a small number of sessions [3, 38]. In comparison to EMDR and IE, IMRS can be more easily applied to a wider range of traumas including emotional abuse and neglect and can effectively address complex emotions such as guilt and shame [3]. For these reasons, the current study examines the effect of IMRS to treat trauma in uED patients.

The first aim of the present study was to explore whether IMRS, when added to a clinical eating disorder treatment, is effective in reducing trauma-related symptoms in uED patients. The second aim was to explore whether treatment of trauma had an effect on the process of weight gain and on eating disorder pathology in general. We investigated this treatment in a group of 10 patients, by using a randomized multiple baseline design with five baseline lengths (6 to 10 weeks) so that we have a higher power of verification of the results because the design controls for time and assessment effects [14]. The variation in baseline length gives the opportunity to distinguish between time effects and effects of the IMRS treatment.

## Method

### Participants

Participants were originally 12 uED patients with PTSD, who underwent inpatient treatment for their eating disorder from February 2017 until July 2019. Mean age was 25.73 (SD 11.09, range 16–58). Two patients dropped out of the study early. One discontinued clinical treatment during the baseline period of the study because she had difficulty with participating in the group; she was diagnosed with an autism spectrum disorder. In a dropout interview she indicated that the upcoming trauma-treatment was a reason for her to try and remain in clinical treatment not a reason to stop clinical treatment. The other stopped after three IMRS sessions. In a dropout interview she indicated that her trauma complaints diminished immediately after the IMRS sessions. However, the sessions consumed so much energy that she felt she had too little energy left to make the most of her clinical eating disorder treatment. She preferred a sequential treatment rather than a parallel treatment. Because this study was a first concept of proof, we have reported on the 10 participants that completed the IMRS treatment. All participants were female and of Dutch nationality. Inclusion criteria were: (1) a BMI between 14 and 16.5; (2) current DSM 5 diagnosis for AN or Other Specified Feeding and Eating Disorder (OSFED); (3) a PTSD diagnosis as defined by DSM-5 and determined with the Structured Clinical Interview DSM (SCID-5) PTSD section [23], and the Clinically Administered PTSD Scale (CAPS-5) interview [61], (4) age between 16 and 65 years; (5) an indication for inpatient treatment; (6) willingness to participate in the study (signed informed consent). Exclusion criteria were: (1) estimated IQ < 80; (2) acute suicide risk; (3) substance dependence; (4) life threatening physical condition; (5) having started with new medication in the 3 months prior to the start of the study; (6) ongoing trauma; (7) a medical history of psychosis, bipolar disorder, or borderline personality disorder.

Of the 72 patients that were clinically admitted in the defined period, 12 met all inclusion criteria. All participants that met the inclusion criteria agreed to participate in the study and provided written consent.

**Fig. 1:**
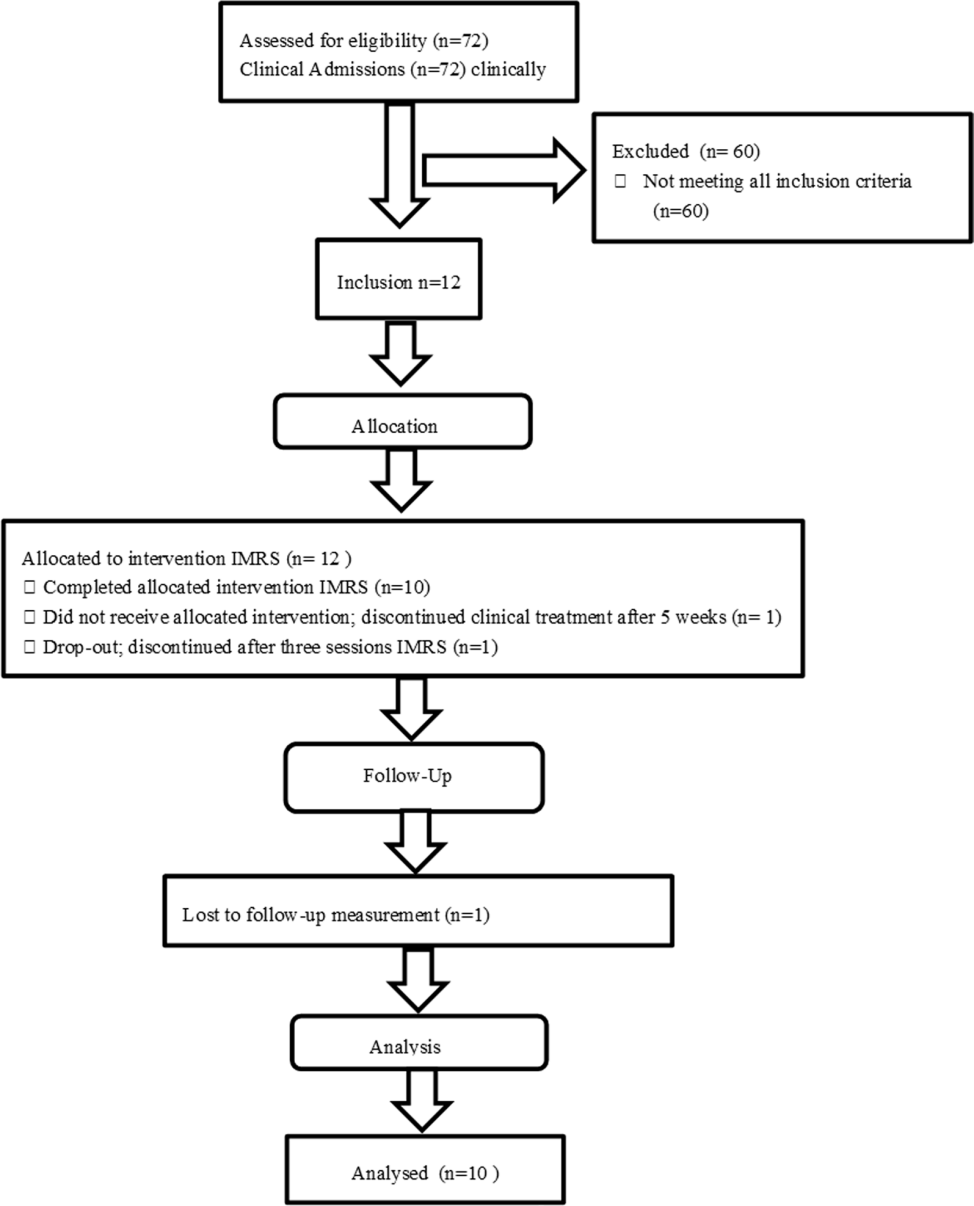
Consort flow diagram

Figure 1 presents a consort flow diagram with eligible patients, excluded patients and those lost to the study.

Table 1 presents the demographic and clinical data of the completers group (N = 10).

**Table 1:** Demographic and clinical data of the completers group (N = 10)

Variable		
Age (mean, SD)	Range 16–58 (n = 10)^+^	26.4 (12)
Gender (number, %)	Female	10 (100%)
Completed educational level (number, %)	Pre-vocational secondary education	3 (30%)
	Secondary vocational education	3 (30%)
	Senior general secondary education	1 (10%)
	Pre-university education	2 (20%)
	University education	1 (10%)
Feeding and eating disorder (number, %)	Anorexia nervosa	9 (90%)
	Other specified feeding and eating disorder	1 (10%)
Body Mass Index, start of the study (mean, SD)	Range 14.9–17.8	15.6 (1.2)
Body Mass Index, start IMRS phase (mean, SD)	Range 14.6–18.4	16.7 (1)
Number of participants per trauma category (LEC-5 categories^++^)	Physical assault (for example, being attacked, hit, slapped, kicked, beaten up)	4
	Sexual assault (rape, attempted rape, made to perform any type of sexual act through force or threat of harm)	5
	Other unwanted or uncomfortable sexual experience	2
	Combat or exposure to a warzone (in the military or as a civilian)	1
	Any other very stressful event or experience	1
Total CAPS-5^+++^ score (mean, SD)	Range Caps total 33–58	44.8 (9.1)

^+^One outlier of age 58. Without outlier Range 16–30, Mean 22.9, SD 4.9

^++^Weathers et al. [60]

^+++^Clinical-administered PTSD scale for DSM-5

### Procedure

All patients who registered with the expertise centre for FED, were seen in an admission interview by a psychologist and a medical doctor, the participants’ families were seen by a family therapist. Next, the SCID-I for DSM-IV-TR was administered to determine the diagnosis of PTSD [27]. Hereafter all the information was discussed in a multidisciplinary meeting where the treatment advice was compiled. During this multidisciplinary meeting, a checklist was used with inclusion criteria for the IMRS study. Patients who met the inclusion criteria received a brief oral explanation and an information letter (including an informed consent form) about the IMRS study in the admission interview. In the information letter the IMRS method was explained, and because the treatment could temporarily cause emotional distress in patients (as do all therapies which focus on working through trauma), patients were fully informed of these effects. After several days, the patient was approached (by the first author) by telephone, asking whether they were willing to participate in the study. After consent was received, they were invited for the CAPS 5, version last month [9]. The CAPS-5 was used because the Dutch version of the SCID-5 was not yet available at that time. With the CAPS-5 the DSM 5 diagnosis was confirmed. After the CAPS 5 the visual analogue scales (VAS) were personalized.

### Design

**Fig. 2:**
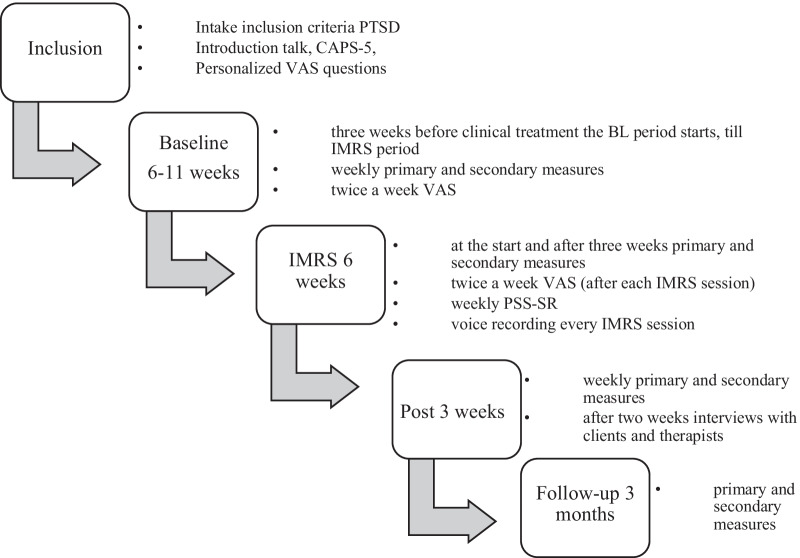
Overview of the design with the different baseline periods and measuring moments

This study was designed as an intervention study during an inpatient treatment program for FED with weekly assessments throughout baseline, therapy, and 3 weeks post-treatment, as well as a follow-up assessment after 3 months (Fig. 2). The participants all started with clinical ED treatment, but different waiting periods (i.e., baseline lengths) were used for the start of IMRS. We utilized a randomized multiple baseline design with five baseline length (6 to 10 weeks). The participants started the trauma treatment 3–7 weeks after commencing their ED treatment. This step-by-step approach design, in which IMRS treatment starts at different times (baseline lengths) for different participants, ensures that the results can be attributed to the IMRS intervention rather than to influences from time and other contextual factors. The participant then serves as a control for herself. Thus, the variation in baseline length gives the opportunity to distinguish between time effects and effects of the IMRS treatment. A randomization schedule for baseline lengths (2 participants per length: 6, 7, 8, 9 and 10 weeks) was determined a priori, and participants were allocated to these randomly predetermined baseline lengths based on inclusion order.

The participants were not informed about the duration of the baseline and were notified 3 days before the start of the IMRS. Due to a miscommunication 1 participant was given the wrong baseline length. This means there was one participant with 9 weeks baseline and three participants with 6 weeks baseline. One participant did not report any traumatic complaints during the screening yet reported more and more PTSD complaints during the first weeks of clinical treatment.^1^ This patient was therefore included in the study during her clinical treatment.

### Power analysis

Although sophisticated statistical techniques to analyse data from case series over subjects have been developed, there is no simple power analysis method developed yet. A sample of n = 10 would yield 80% power to detect a large effect size (Cohen’s d > 1) with a paired t-test at a two-tailed significance level of 0.05. Such an effect size is reasonable to expect given the pooled pre-post effect size of *g* = 1.48; 95% CI = [1.14; 1.82] for IMRS for PTSD [38].

### Treatments and therapists

The inpatient treatment program for FED consists of 5 days of therapy with overnight stays and weekends at home. The program used cognitive behavioural change methods and focused on increasing weight and on the factors that sustain the eating disorder for the patient. Three main meals and three in-between meals a day were supervised. Beside the meals, the program consisted of the following components: (1) One hour of ‘cognitive behavioural therapy’ (emphasis on maintaining factors of the eating disorder in the present and future, (2) two hours of ‘eating behaviour’ (aimed at normalizing eating patterns), (3) two hours of ‘body and movement-oriented therapy’ (focused on the over-evaluation of body, movement behaviour and emotion regulation [19], (4) one hour of psychoeducation, (5) one hour of ‘progress and goal meeting’, (6) 75 min of ‘psychotherapy’, and (7) two hours of ‘activity guidance’ (focused on expanding other areas of life,work, study, friendships, hobbies, sports; with the option to spend 1 h on creative work). A parent and partner group was offered five times during clinical admission (psychoeducation about eating disorders, opportunity for support, recognition, understanding and advice).

The investigational treatment was IMRS. IMRS is a psychological treatment for processing traumatic experiences [4, 5, 44, 53]. IMRS was given in addition to the regular inpatient treatment program for FED. IMRS treatment consisted of 12 IMRS sessions of 90 min each, offered in 6 consecutive weeks. IMRS aims to change the meaning of traumatic experiences by experiencing imagined interventions that correct the dysfunctional emotional and interpersonal meanings attached to the trauma. In IMRS, the patient imagines the start of the traumatic event (to activate the trauma memory). When there is enough emotional activation (usually at the hotspot), the rescripting starts. The therapist, and later the patient (from their current perspective), then rescripts the traumatic experience to provide a more desirable outcome, all the while imagining this new script as lively as possible. This leads to change of maladaptive beliefs, more control over images, and improved possibilities to reassure oneself [34]. This way, the patient does not have to relive the full trauma in all its details. It is important, in this respect, that the rescripted outcome contains new and unexpected information for the patient, so that a lasting change of memory is created [22].

The first IMRS session was a preparatory session, in which the therapeutic alliance was formed, the rationale and treatment were explained, and a list of experienced traumas was established. The therapist and patient discussed the order in which they would address the traumas. Also, current living circumstances were checked (to check whether there was enough distance from the perpetrator and enough safety to conduct trauma processing). This first session ended with a pilot IMRS (using a mildly negative childhood memory) to familiarize the patient with the IMRS technique, and with a session evaluation. Patients were instructed to read the IMRS explanation hand-outs and to reread the list of trauma-themes and change items and/or order if applicable before the following IMRS session.

Within each consecutive session, the following sequential steps were followed:

1. Check for intrusions, nightmares, and emotions since the previous session. Discuss how the previous session affected the patient;
2. Agree upon which trauma theme to start with;
3. Have patient close eyes and retrieve traumatic memory;
4. Therapist (session 1–6) or adult patient (session 7–12) steps into the image;
5. Therapist or adult patient intervenes, and patients imagines this intervention;
6. Check whether the imagined situation is effectively under control and the child’s needs are met;
7. If the way of intervening was not successful: rewind and start again;
8. Stop when patient, from the point of view of the child, says ‘it is okay’;
9. Evaluate the rescripting (rescript another memory if time allows);
10. Evaluate the session;
11. Assign homework: review trauma list (for order and needs or wishes to address traumas).

For a more detailed treatment description see Raabe et al. [44]. The IMRS sessions were given by postdoctoral trained registered psychologists (two health psychologists, two psychotherapists and one clinical psychologist). Therapists were trained during a one-day workshop by Arnoud Arntz.

The therapists attended weekly group supervision (60 min). These were led by the first author with the option of consulting the fourth author by telephone or email.

### Treatment integrity

To assure that IMRS was carried out as designed, the treatment integrity was assessed. Treatment sessions were audio-recorded, and the adherence was rated by two trained and independent master-level doctoral psychology students, with the use of the Imagery Rescripting Therapist Adherence and Competence Scale [45]. The students were trained by the first author. Besides that, 4 IMRS sessions were rated by both students to assess interrater reliability [43]. The mean of the treatment adherence of all elements of IMRS was 0.80 (SD 0.071 (0 = the therapist didn’t demonstrate the intervention, 1 = the therapist demonstrated the intervention. This means that 80% of the prescribed elements was detected. Interrater reliability was good (intraclass correlation coefficient [ICC] = 0.66, *p* < 0.001 [adherence]).

### Instruments

The main primary outcome measure was the PTSD Scale-Self Report for DSM-5 (PSS-SR), which was used to assess the level of PTSD-related symptoms according to DSM-IV [25]. The scale assesses the frequency of the trauma related symptoms during the last week (range 0–51), with a 4-point scale (3 = very often, always, 2 = often, 1 = sometimes, 0 = never). The completed scores are summed, a higher score reflecting more PTSD symptoms. Sin et al. [52] showed that the optimal cut-off point for the PSS-SR is 14 and the scale has high internal consistency and validity. Cronbach’s alpha in the present study was 0.87.

The second group of primary outcomes were core emotional problems and beliefs assessed using Visual Analogue Scales (VAS). The respondents indicated to which extent they experienced an item (not at all—extremely) on a 100 mm line with two anchors. The following ‘negative emotions’ items were presented (1) to which extent did you experience rage in the past 3 days, (2) to which extent did you experience guilt in the past 3 days, (3) to which extent did you experience shame in the past 3 days, (4) to which extent did you experience disgust in the past 3 days. In addition, three to four personalized ‘dysfunctional self-beliefs’ and ‘dysfunctional body-beliefs’ VAS scales with negative thoughts about the self and the body were added. These were: to which extent did you suffer from your personalized negative thoughts about the self/body in the past 3 days? (not at all—extremely). An example of such an idiosyncratic VAS is ‘I’m fat’. The VAS scales were collected twice per week during baseline period, after each IMRS session, twice per week during the follow-up and once during the follow-up measure. We derived three composite scores from the VASs; a negative emotion score (the average of the emotion VASs), a dysfunctional self-belief score (the average of the idiosyncratic beliefs about the self) and a dysfunctional body-belief score (the average of the idiosyncratic beliefs about the body).

The second outcome measures consisted of the Post-Traumatic Cognitions Inventory (PTCI), the Body Mass Index (BMI), the Eating Disorder Evaluation-Questionnaire (EDE-Q) and the Difficulties in Emotion Regulation Scale (DERS). These parameters were administered at fixed timepoints (weekly during baseline period, at the start, middle and end of the IMRS, weekly during follow-up and once during follow-up measure).

The Post-Traumatic Cognitions Inventory (PTCI) was used to measure trauma-related cognitions. An example of a question is ‘I am a weak person’ or ‘The world is a dangerous place’. It assesses the frequency of the trauma-related cognitions during the last week (range 0–51), with a 4-point scale (0 = never, 1 = sometimes, 2 = often, 3 = very often, always). The scores are added together, the higher the score the more trauma-related cognitions. Van Emmerik et al. [57] and Foa et al. [24] found a high internal consistency and high 2-week test–retest reliability of the PTCI. Cronbach’s alpha of the total score in the present study was 0.91.

BMI is a relative weight measure and is calculated as follows: body weight in kg/body height in m^2^. During inpatient treatment, height is measured once during initial screening, and weight is measured at least two times a week. We asked patients for permission to use the height and weekly weight information from their medical files.

The Eating Disorder Evaluation-Questionnaire (EDE-Q 6.0) is a 28-item self-report questionnaire which is used to measures core attitudinal features of eating disorders for the past 28 days, and the frequency of core eating disorder behaviour from the previous 7 days [21]. The EDE-Q includes features of eating disorders and specific behavioural symptoms. An example of a feature is ‘eating concern’ and of a specific behavioural symptom ‘self-induced vomiting’. The EDE-Q demonstrated reliability of scores, but additional research is needed to generalize these findings [8]. Cronbach’s alpha of the total score in the present study was 0.89.

The Difficulties in Emotion Regulation Scale (DERS) is 36 item self-report questionnaire which is used to measure difficulties with emotion regulation [29, 40]. An example of a question is ‘I know what my feelings are’ or ‘When I’m upset, I feel ashamed about it’. The DERS has good test–retest reliability [29] and high internal consistency within clinical patients [26]. Cronbach’s alpha in the present study was 0.92.

### Statistical analysis

Mixed regression was used for the primary (PSS-SR, VAS) and secondary (PTCI, BMI, EDE-Q, DERS) quantitative outcome parameters, to assess differences between treatment, post-treatment and follow-up measures compared to baseline in average scores and linear change. Fixed variables were a dummy to indicate treatment, a dummy indicating the post-treatment and a dummy indicating the follow-up measure (so that baseline is the reference), and time by phase interactions represented by a centred linear time effect within each phase [58]. Random variables were a random intercept to capture between subject outcome variation, plus Autoregressive Moving Average Model (ARMA1.1) for the within-subject covariance structure of the repeated part. ARMA1.1 had the best fit for all the questionnaires, except for the VAS scales, in which case we used Autoregressive model (AR1). Given their skewed distributions, the transformed (101—raw score in mm) outcome measures ‘VAS body’ and ‘VAS belief’ were analysed by Generalized Linear Mixed Models with a gamma distribution and log-link, which is suitable for (very) skewed distributions. We assessed the full model with all predictors.

The effect sizes of the changes in dependent variables were expressed in terms of Cohen’s d based on the difference between the estimated means from the mixed regression as numerator and baseline SD as denominator: d(moment x) = (M(baseline)  − M(moment x)/SD(baseline) [6, 58].

## Results

### PSS-SR

**Fig. 3:**
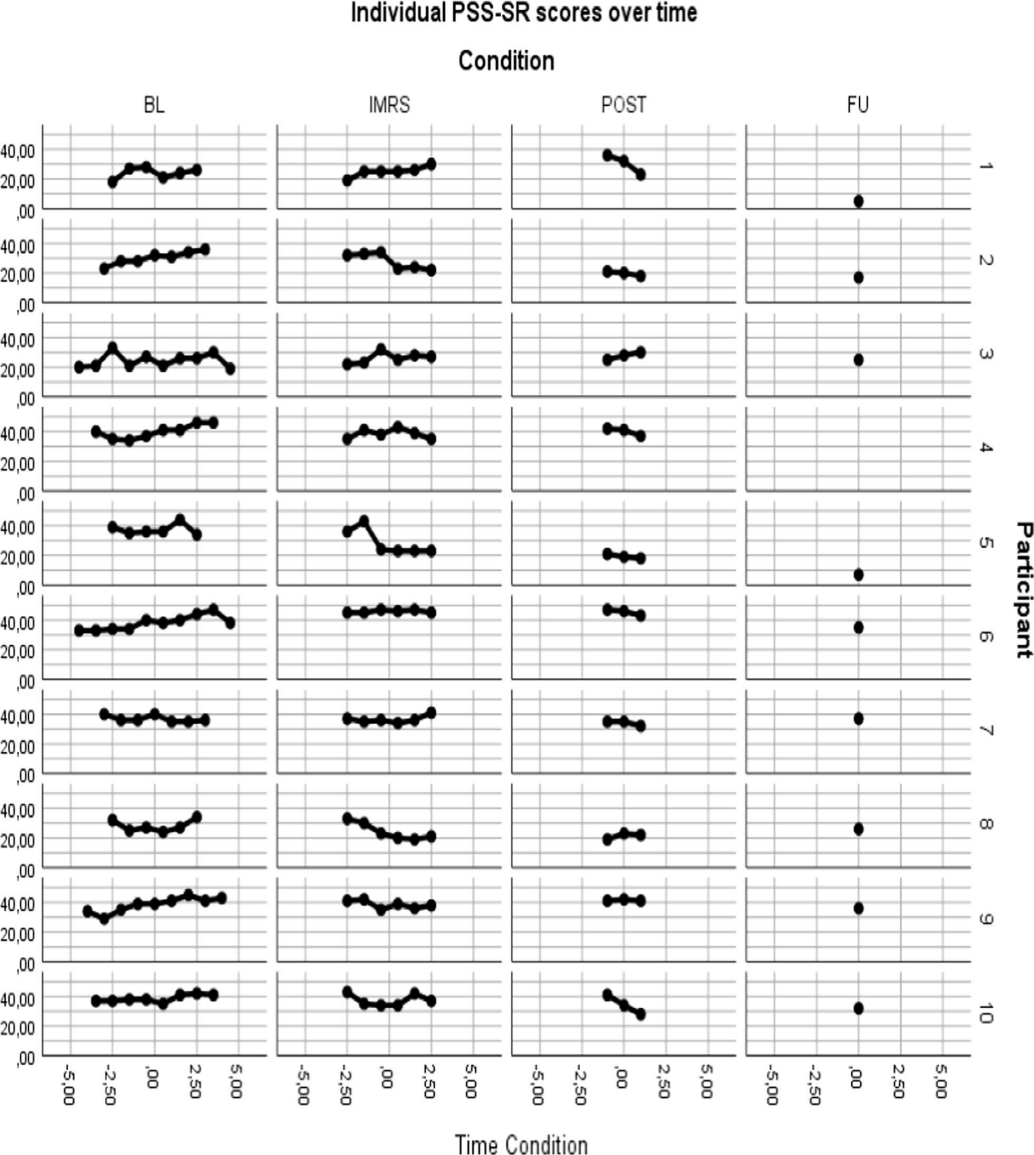
Individual PSS-SR scores over time

In Fig. 3, individual participants scores on the PSS-SR are shown. During baseline, 6 out of 10 participants show a slight increase in PTSD symptoms. During the IMRS this changes, with 4 participants showing a decrease in PTSD symptoms, while 3 participants show an increase in PTSD symptoms and 3 participants showing stable symptoms. During post-treatment/FU, 7 participants showed reductions compared to mean baseline scores. None showed long-term deterioration.

Table 2 presents the results of the mixed regression analysis. Note that the beta’s (and t-tests, and effect sizes) of all phases, except follow-up, represent the main effect at (within-condition centred) time = 0, which is halfway the pertinent phase. Results showed no significant changes in PTSD symptoms during the baseline and treatment phase, but a significant reduction during the post-treatment phase. Furthermore, PTSD symptoms at follow-up were significantly lower compared to baseline, with a very large effect size [51].

**Table 2:** Results mixed regression analyses

	Parameter	Beta	Std. error	df	t	*p*	Effect size Cohen’s d
PSS-SR^+^	Intercept	33.87	2.45	12.19	13.83	< 0.001	
	Treatment	− 1.06	1.51	24.21	− 0.70	0.491	0.64
	Post-treatment	− 2.72	2.18	14.43	− 1.25	0.231	0.93
	3-months follow-up	− 8.70	2.52	15.92	− 3.45	0.003	1.53
	Time within baseline	0.58	0.30	41.34	1.94	0.060	
	Time within treatment	− 0.57	0.38	48.96	− 1.50	0.140	
	Time within post-treatment	− 1.79	0.87	144.41	− 2.06	0.041	
VAS^++^ Negative emotions	Intercept	71.73	6.45	9.98	11.12	< 0.001	
	Treatment	1.91	2.89	34.72	0.66	0.512	1.03
	Post-treatment	− 4.04	4.20	33.75	− 0.96	0.343	1.08
	3-months follow-up	− 10.42	4.94	57.69	− 2.11	0.039	1.39
	Time within baseline	2.97	0.75	44.07	3.98	< 0.001	
	Time within treatment	− 2.17	1.03	58.12	− 2.10	0.040	
	Time within post-treatment	− 1.35	2.22	286.01	− 0.61	0.544	
VAS^++^ Self-belief^a^	Intercept	2.14	0.34		6.22	< 0.001	
	Treatment	0.24	0.14		1.67	0.099	1.15
	Post-treatment	0.52	0.22		2.37	0.20	0.99
	3-months follow-up	0.93	0.25		2.86	0.005	2.35
	Time within baseline	− 0.19	0.04	89	− 5.04	< 0.001	
	Time within treatment	0.13	0.05	104	2.50	0.014	
	Time within post-treatment	− 0.02	0.20	293	− 0.09	0.932	
VAS^++^ Body-belief^a^	Intercept	1.37	0.37		3.74	0.004	
	Treatment	0.08	0.12		0.65	0.518	1.16
	Post-treatment	0.46	0.18		2.51	0.014	1.41
	3-months follow-up	1.45	0.26		5.67	< 0.001	3.79
	Time within baseline	− 0.13	0.03	70	− 4.22	< 0.001	
	Time within treatment	0.09	0.04	86	2.06	0.043	
	Time within post-treatment	− 0.01	0.15	293	− 0.04	0.972	
PTCI^+^	Intercept	179.42	5.82	13.37	30.81	< 0.001	
	Treatment	6.57	4.35	42.08	1.51	0.138	1.80
	Post-treatment	− 8.13	6.15	15.98	− 1.32	0.205	2.98
	3-months follow-up	− 15.35	7.08	18.88	− 2.17	0.043	4.00
	Time within baseline	3.77	0.93	40.68	4.04	< 0.001	
	Time within treatment	− 4.13	1.84	85.34	− 2.24	0.028	
	Time within post-treatment	− 0.35	2.49	107.96	− 0.14	0.887	
BMI^+^	Intercept	16.08	0.49	9.84	32.82	< 0.001	
	Treatment	0.95	0.23	125.25	4.20	< 0.001	− 1.75
	Post-treatment	1.83	0.38	108.79	4.78	< 0.001	− 2.52
	3-months follow-up	2.34	0.42	104.87	5.52	< 0.001	− 3.80
	Time within baseline	0.14	0.05	127.90	3.03	< 0.001	
	Time within treatment	0.24	0.08	122.70	3.05	0.003	
	Time within post-treatment	− 0.03	0.09	118.76	− 0.32	0.752	
EDE-Q^+^	Intercept	4.51	0.40	10.74	11.41	< 0.001	
	Treatment	− 0.07	0.20	11.78	− 0.34	0.739	0.08
	Post-treatment	− 0.14	0.28	4.83	− 0.52	0.627	0.29
	3-months follow-up	− 0.75	0.31	6.35	− 2.42	0.050	1.45
	Time within baseline	− 0.03	0.05	17.81	− 0.71	0.486	
	Time within treatment	− 0.03	0.09	72.52	− 0.32	0.752	
	Time within post-treatment	− 0.12	0.12	118.82	− 0.97	0.333	
DERS^+^	Intercept	117.41	5.32	10.94	22.08	< 0.001	
	Treatment	0.27	2.56	33.71	0.11	0.917	0.77
	Post-treatment	− 1.86	3.60	9.33	− 0.52	0.617	0.97
	3-months follow-up	− 13.19	4.27	10.90	− 3.09	0.010	2.41
	Time within baseline	0.96	0.53	27.62	1.81	0.081	
	Time within treatment	− 1.13	1.12	87.76	− 1.01	0.316	
	Time within post-treatment	− 0.82	1.57	111.70	− 0.52	0.602	

^a^Analyzed by mixed gamma regression with a log link, after inversing raw scores by 101-raw score (in mm.). This implies that beta’s are in transformed scale, and that positive time effects denote improvement

^+^^,^^++^Time-within-Condition: 0 for measurements outside the condition, centred time (with a week as unit) for measurements within condition (^+^e.g., − 3, − 2, − 2, − 1, 0, 1, 2, 3 for a 6-week condition; ^++^e.g., − 3, − 2.5, − 2, − 1.5, − 1, − 0.5, 0, 0.5, 1, 1.5, 2, 2.5, 3 for 2 weekly measures in a 6 week condition)

### Core emotional problems and beliefs

**Fig. 4:**
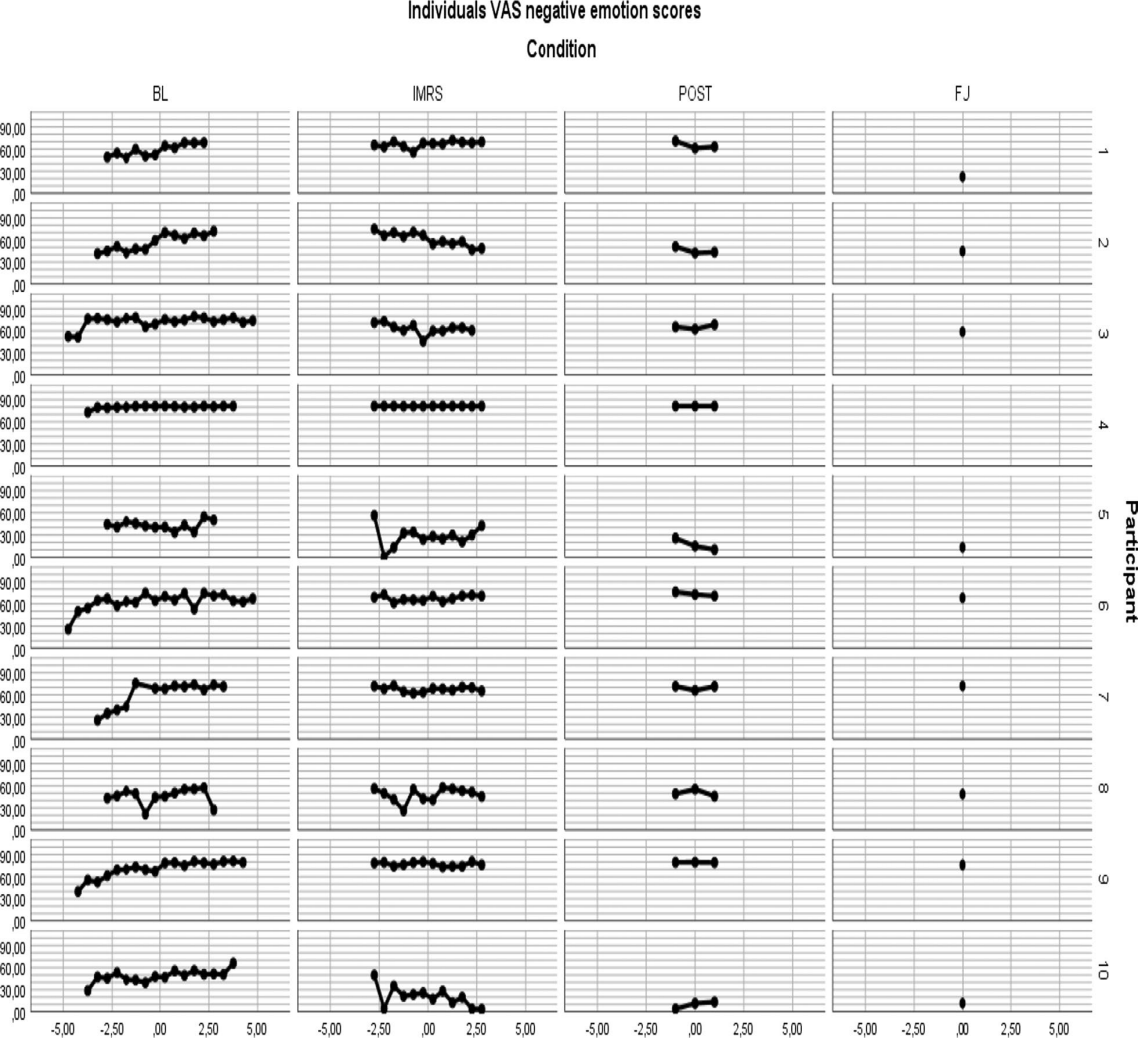
Individual VAS negative emotions scores over time

In Fig. 4 (“Appendix”) the individual scores on VAS 1 to 4 (the negative emotions) are shown.

**Fig. 5:**
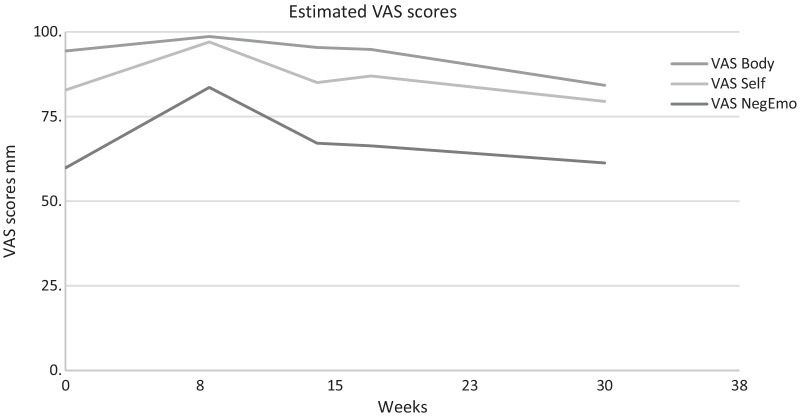
Means VAS negative emotions, self- beliefs, body-beliefs per condition. Baseline mean is set at 8 weeks. VAS Negative Emotion is analysed with LMM. VAS Body and VAS Self were analysed with GLMM, gamma distribution and transformed into the original scale

Results show that there was a significant increase of core negative emotions and beliefs during the baseline phase, and a significant decrease during the treatment phase. There were no significant changes between core negative emotions and beliefs at treatment and post-treatment phases, compared to the baseline phase, except for the post-treatment body beliefs, which were significantly lower compared to baseline. No significant main effects of treatment and post-treatment on negative emotions were found (but note that these effects are estimated halfway the pertinent phase). Core negative emotions and beliefs at 3-months follow-up were significantly lower compared to baseline, with a very large effect size (Table 2) [51]. Figure 5 shows the total mean scores of the participants on VAS negative emotions, VAS self-beliefs (the negative idiosyncratic beliefs about the self) and VAS body-beliefs (the idiosyncratic belief about the body emotions).

### PTCI

Mixed regression analysis showed no significant effect of IMRS on PTCI scores during treatment and post-treatment, compared to baseline scores. A significant effect was found compared to baseline at 3-months follow-up, effect size was very high (see Table 2). During baseline the PTCI scores increased significantly. During treatment the PTCI scores decreased significantly.

### BMI

Mixed regression analysis showed that, compared to baseline, BMI was significantly lower at all phases, with very large effect sizes (see Table 2). The time-within-baseline and time-within-treatment scores also showed significantly increased levels of BMI, while there was no significant change within the post-treatment phase.

### EDE-Q and DERS

Regarding eating disorder features and emotion regulation problems, results showed no differences between the baseline scores and the treatment and post-treatment scores, while the follow-up scores were significantly lower compared to the baseline scores, with a high effect size (see Table 2). During the separate phases, there was no significant change in eating disorder features or emotion regulation problems.

### Serious adverse events

One participant was diagnosed with a conversion disorder and she indicated that she had psychotic symptoms during inclusion at the start of the study. Since these psychotic symptoms were directly related to the trauma to be treated, the participant was not excluded from the study. Because these psychotic symptoms increased during treatment, an anti-psychotic was started. The conversion complaints worsened during the IMRS, but not to the extent that the IMRS had to be stopped. Loss of contact with one's own body during the IMRS sessions also emerged in two other participants, but these participants were able to complete the trauma-treatment. Thus, while some symptoms (temporarily) increased, no serious adverse events took place (i.e., events that necessitated hospitalization and/or were life threatening).

### Feedback from participants

Two weeks after the IMRS all participants and therapists were asked about their experiences with IMRS by means of an interview. It was noticeable in the feedback from the therapists that they found IMRS a pleasant method to work with in underweight patients.

Participating patients reported experiencing sufficient emotions and concentration to engage in IMRS. More detailed and in-depth information from these interviews will be described in a separate article.

## Discussion

As far as we know, this is the first study that investigated if treating PTSD with IMRS is possible in uED patients. We used a randomized multiple baseline case series design with 10 participants. Mixed regression analyses revealed that there was a significant effect on the follow-up measure compared to baseline on all outcomes. On the primary outcome measures we found a significant reduction of severity of the PTSD symptoms, on average emerging at post-treatment, as shown by the clear progress on the PSS-SR. Furthermore, a significant decrease emerged in negative emotions (anger, shame, guilt, disgust) and personal negative beliefs about the self and the body as shown by the VAS scores at the follow-up measure. The secondary outcome measures showed a reduction of trauma-related thoughts and beliefs and a reduction in difficulties with emotion regulation. In addition, we found that the eating disorder symptoms also decreased as shown by the EDEQ while levels of Body Mass Index increased.

When looking at the pattern in the baseline period, we notice that the scores of the trauma related cognitions (measured by the PTCI) and the core emotional problems and core beliefs (measured by the VAS) worsened significantly. PTSD-symptoms (PSS-SR) and emotion regulation (DERS) also worsened during baseline, albeit not significantly. The EDEQ did not change significantly during baseline. Looking further at the pattern, we notice that the PSS-SR, the EDEQ and the DERS only improved during post-treatment/at follow-up measurement, while the VAS scores and the PTCI showed improvement during the IMRS. In sum, participants showed a pattern of deterioration on most outcomes during baseline, when the weight-gain program started, while slowly improving after IMRS started, with the earliest change achieved in core emotions and cognitions, after which in the long-term large changes were achieved.

### Interpretation and implications

These findings are clinically important. They show that in this challenging patient group PTSD symptoms can be reduced by IMRS but that it takes time. The finding that IMRS was effective is in line with a lot of treatment studies about the application of IMRS in different disorders [3, 38]. However, usually IMRS has immediate effects, and the present finding that initial responses are weak might be specific for this patient population – although the worsening of problems during baseline stopped with the start of IMRS. The present results also indicate that there was sufficient experience of emotions and cognitive functioning to do trauma-treatment in these uED-patients.

The pattern during the baseline period fits clinical experience that if BMI increases and/or eating disorder patients start clinical treatment their trauma-related problems worsen and make recovery from the eating disorder more difficult. It is likely that the increase in weight and the reduced possibilities of using eating disorder behaviour as distractor can explain why patients experience more negative emotions and are more aware of ED as well as non-ED cognitions.

The pattern that the PSS-SR, the EDE-Q and the DERS only improved during the follow-up measurement, while the mean VASs scores and the PTCI showed improvement during the IMRS, may indicate something about the possible mechanisms of change. First, we see change in core emotions, core beliefs, and trauma-related cognitions, then we see change in the pathology of the eating disorder and the PTSD. The change in difficulty with emotion regulation comes afterwards. This is in concurrence with the supposed mechanism of IMRS: change of meaning (i.e., affective, and cognitive meanings) of trauma representation [3]. Our finding that emotion regulation improves is in line with Raabe et al. [46] who showed that IMRS also had an effect on emotion regulation. This gives rise to the hypothesis that changing the meaning of traumatic experiences, in uED patients, may help them regulate emotions.

### Limitations of the present study

First, this study was a proof-of-concept study, in which we only analysed completers. As a result, the data of two people were not included in the analysis, which can give a distorted picture of overall effectiveness.

Secondly, it is important to mention that no distinction was made in the inclusion criteria regarding single versus multiple and/or long-lasting trauma. This might influence the effects because multiple/long lasting trauma is associated with many other psychological problems [20]. In the current study, one participant had two single trauma’s and all the other participants had multiple/long lasting childhood traumas.

Thirdly we were not able to control for confounders because of the small sample. For future research we recommend using a larger sample and to control for confounders such as; single or multiple traumas, type of trauma and for trauma treatment history (already had previous trauma treatments or not).

Fourthly, we did not differentiate in number of sessions using the Imagery Rescripting (IREM) 12 session protocol [4]. For some participants this was more than enough, while others indicated that 12 sessions were a good start but it would have been nice if a few more could have been added. This was specifically pointed out by several patients with very long-lasting multiple traumas.

Fifth, we chose to use IMRS for the treatment of PTSD because it had a superior effect in diminishing complex trauma-related emotions compared to imaginal exposure in one study [7] and because it was considered better tolerable for patients than IE, resulting in a lower dropout rate [44]. Boterhoven de Haan et al. [10] showed a low dropout rate for both EMDR and IMRS. The current study contributes to evidence that IMRS is as an effective trauma-treatment. However, which trauma-treatment is especially effective and tolerable in patients with uED and PTSD is an issue for further research.

### Strengths of the present study

The first strength is the fact that the cases were highly complicated with severe eating disorders for which clinical treatment was required: Patients were seriously underweight at the start of the study (mean BMI 15.6), and at start of the IMRS treatment (mean BMI 16.8). PTSD severity was high with an average baseline score of 45.6 on the CAPS [9]. There was one index trauma that involved death or threatened death, 4 involved actual or threatened serious injury and 5 involved actual or threatened sexual violence. Most of the trauma experience were in the category of sexual abuse (e.g., rape, incest), in the category non-sexual child abuse (physical abuse, emotional/psychological abuse), or in the category emotional neglect. Five participants used medication. The findings indicate that IMRS works for this very complex group of patients, which makes it plausible that it will also work for less complex cases.

The second strength of this study is the randomization over multiple baseline lengths, which increases the certainty with which causal conclusions can be drawn: it is highly unlikely that the mere passage of time, or the repeated assessments, caused the improvements, given the worsening of many symptoms during baseline. A sample size of N = 10 seems very small compared to a RCT with much more participants. However, a randomized multiple baseline case series study can be a very powerful way to assess the effectiveness of a treatment and has high clinical validity because of its high resemblance to clinical practice [42].

### Recommendations

The present study was the first study we know of that investigated the treatment of PTSD in uED patients. Because of the different opinions on the impossibility of treatment of trauma in underweight patients we performed a small proof of concept study, with a randomized multiple baseline case series design. This study clearly shows evidence for strong effects of IMRS in decreasing PTSD symptoms, when treating uED patients, without observable negative effect on eating disorder treatment. These results give rise to setting up an RCT to further substantiate the possibility and effectiveness of trauma processing early in treatment for this group of uED patients with comorbid PTSD. Another issue for further study is the investigation of the reasons for the initially slow rate of change. For instance, it could be investigated whether this is attributable to weight, or to specific characteristics of these patients independent of their weight. An important issue for future RCTs is to test what the optimal phase in ED-treatment is to do trauma processing, and perhaps even more importantly, what the optimal phase is for whom. Although we found positive effects for trauma processing in the early phase of ED treatment in the current study, it is possible that trauma processing earlier or later in ED-treatment has even better effects.

Besides this, it is advisable to investigate whether the pattern of increased PTSD-symptoms and other problems during baseline recurs and to investigate the cause.

## Conclusion

Summarizing, this study clearly shows that treating PTSD symptoms with IMRS in uED patients is effective. IMRS in this patient group was shown to have a positive impact on the reduction of PTSD symptoms and on the emotional experience of anger, shame, guilt, and disgust. Also, negative cognitions about the world, about the self and the body decreased. Difficulties with emotion regulation reduced. Importantly, we found that eating disorder pathology decreased at follow-up and BMI increased, which indicates that the IMRS treatment did not disturb the eating disorder treatment. There was no negative effect for trauma-treatment on the eating disorder pathology during the treatment. The present study raises hopes for traumatized underweight ED-patients for whom trauma treatment was traditionally unobtainable.

## Data Availability

Reasonable requests for data will be considered by the authors under the condition that the European Data Protective is guaranteed for these sensitive patient data, and an appropriate analytic plan is included.

## Appendix

See Fig. 4.

## Abbreviations

AN: Anorexia nervosa
AR: Autoregressive model
ARMA: Autoregressive moving average model
BMI: Body Mass Index
CAPS: Clinically Administered PTSD Scale
DERS: Difficulties in Emotion Regulation Scale
DSM: Diagnostic and Statistical Manual
EMDR: Eye Movement Desensitization and Reprocessing
ED: Eating Disorder
EDE-Q: Eating Disorder Evaluation-Questionnaire
FED: Feeding and Eating Disorder
FU: Follow up
ICC: Intraclass Correlation Coefficient
IE: Imaginal Exposure
IMRS: Imagery Rescripting
IREM: Imagery Rescripting Eye Movement Desensitization and Reprocessing
OSFED: Other Specified Feeding and Eating Disorder
PTCI: Post-Traumatic Cognitions Inventory
PSS-SR: PTSD Scale-Self Report for DSM-5
PTSD: Post Traumatic Stress Disorder
RCT: Randomized clinical trial
SCID: Structured Clinical Interview DSM
SD: Standard deviation
STAIR: Skill Training in Affective Regulation
VAS: Visual Analogue Scales
uED: Underweight Eating Disorders

## Footnotes

1 As Zimmerman and Mattia [63] indicate, some patients feel uncomfortable to share their trauma complaints at the start of treatment. They advise to keep on evaluating on trauma complaints during treatment.
